# Cancer Incidence Trends in the Oil Shale Industrial Region in Estonia

**DOI:** 10.3390/ijerph17113833

**Published:** 2020-05-28

**Authors:** Jane Idavain, Katrin Lang, Jelena Tomasova, Aavo Lang, Hans Orru

**Affiliations:** 1 Institute of Family Medicine and Public Health, Faculty of Medicine, University of Tartu, Ravila 19, 50411 Tartu, Estonia; Katrin.Lang@ut.ee (K.L.); Hans.Orru@ut.ee (H.O.); 2 Department of Health Statistics, National Institute for Health Development, Hiiu 42, 11619 Tallinn, Estonia; 3 Estonian Health Board, Paldiski mnt 81, 10617 Tallinn, Estonia; Jelena.Tomasova@terviseamet.ee; 4 Institute of Biomedicine and Translational Medicine, Faculty of Medicine, University of Tartu, Ravila 19, 50411 Tartu, Estonia; Aavo.Lang@ut.ee; 5 Department of Public Health and Clinical Medicine, Umea University, SE-901 87 Umea, Sweden

**Keywords:** oil shale, lung cancer, air pollution, occupational health

## Abstract

Large oil shale resources are found in Eastern Estonia, where the mineral resource is mined, excavated, and used for electricity generation and shale oil extraction. During industrial activities in the last 100 years, pollutants have been emitted in large amounts, some of which are toxic and carcinogenic. The current study aims to analyse time trends in cancer incidence in the oil shale industry-affected areas and compare them with overall cancer incidence rates and trends in Estonia. We analysed Estonian Cancer Registry data on selected cancer sites that have been previously indicated to have relationships with industrial activities like oil shale extraction. We included lung cancer, kidney cancer, urinary bladder cancer, leukaemia, breast cancer, and non-Hodgkin’s lymphoma. A statistically significantly higher lung cancer age-standardized incidence rate (ASIR) was found during the study period (1992—2015) only in males in the oil shale areas as compared to males in Estonia overall: 133.6 and 95.5 per 100,000, respectively. However, there appeared to be a statistically significant (*p* < 0.05) decrease in the lung cancer ASIR in males in the oil shale areas (overall decrease 28.9%), whereas at the same time, there was a significant increase (*p* < 0.05) in non-oil shale areas (13.3%) and in Estonia overall (1.5%). Other cancer sites did not show higher ASIRs in the oil shale industrial areas compared to other areas in Estonia. Possible explanations could be improved environmental quality, socio-economic factors, and other morbidities.

## 1. Introduction

Cancer is one of the leading causes of death in the world and in Europe [1]. In 2018, there were 18.1 million new cancer cases worldwide. Lung cancer is the most diagnosed cancer site (11.6% of the total cases), closely followed by female breast cancer (11.6%), prostate cancer (7.1%), and colorectal cancer (6.1%) [2]. Cancer incidence is high in Eastern Europe, including Estonia, for some of the neoplasms like lung, stomach, and pancreas cancer [2,3]. Higher incidence rates of those cancers in the region have been associated with smoking, low per capita GDP and high carbohydrate consumption [4]. Since the collapse of the Soviet Union, cancer incidence has increased in North-Eastern Europe and Estonia [1,5] due to multiple aetiologies [6].

Cancers are multifactorial diseases, and according to the International Agency for Research on Cancer (IARC), air pollution plays a significant role in the development of cancer [7]. Polluted air, soil, and water are a problem in most industrial areas all over the world [8]. According to the World Health Organization definition, those areas are called “industrially contaminated sites” and are defined as: “Areas hosting or having hosted human activities which have produced or might produce environmental contamination of soil, surface or groundwater, air, food-chain, resulting or being able to result in human health impacts”.

Since the beginning of the 20th century, Ida-Viru County in North-Eastern Estonia has been the main industrial area of the country, focussing on oil shale production [9]. Oil shale is an organic-rich sedimentary rock containing kerogen that has been mined and excavated there and used for electricity generation and shale oil extraction in chemical industry. The usage was greatest during the 1980s (up to 30 million tonnes per year); subsequent to the collapse of the Soviet Union, it decreased gradually until 1999 (~9.5 million tonnes per year) and has been increasing since (up to 15 million tonnes per year currently) [10,11].

Oil shale mining, shale oil production, and electricity generation processing emit large amounts of particulate matter (PM_10_), sulphur dioxide (SO_2_), and nitrogen oxides (NO_x_), as well as several other industrial pollutants like phenols, and benzene released in oil extraction and other trace elements [12]. Moreover, workers in the mines and in the chemical industries are exposed to health hazards due to the toxicity of oil shale and its derivates [13]. Environmental pollution (atmosphere, soil, groundwater) in the area was specially alarming during 1980s and 1990s due to unprotected hazardous landfills containing ash from oil shale combustion, and semi-coke and retorting waste from refining [14]. Although the environmental quality in the region has improved, and the volumes of excavation are still lower than in the 1980s, the health impacts remain [15,16].

During different periods, studies on the environmental and health impacts of oil shale production and shale oil extraction have been conducted. Regarding cancer, higher lung and stomach cancer rates were recorded in the industrial areas in North-Eastern Estonia in the 1970s [17]. Later, during the 1980s, Etlin et al. [18] conducted several epidemiological studies in the industrial area in Ida-Viru County to investigate cancer sites such as lung, stomach, and skin cancer. However, none of the cancer sites showed significantly (*p* > 0.05) higher incidence in the industrial area compared to the Estonian average. These results, though, may be attributable to methodological shortcomings of the studies, such as poor exposure information or incomplete/missing data on outcomes.

In general, in the past, incidence rates for all cancers combined and specifically lung cancer have been a little higher in North-Eastern Estonia (Ida-Viru County and the towns of Narva and Kohtla-Järve) for males when compared with the rest of Estonia [19]. In the last decade, the deaths due to malignant neoplasms in North-Eastern Estonia have been higher than on average in Estonia [20].

According to IARC, some petroleum products, such as gasoline and heavy fuel oils (including shale oil), contain toxic substances such as benzene and polycyclic aromatic hydrocarbons (PAHs) [21]. These products have been released into the outdoor air in the oil-shale region [16], and even higher exposures have happened in workplaces [22,23]. Benzene, as well as many of the polycyclic aromatic hydrocarbons, is classified as carcinogenic [24,25], and shale oil itself has shown indications of mutagenicity [26].

Recently, increasing evidence has shown associations between living near industrially contaminated sites or being exposed to industrial air pollution and increased total cancer incidence and mortality [27,28,29,30]. Although the main focus has been on lung cancer [28,29,31,32], the increased risks have also been documented for kidney [33,34], bladder [31,32,33,34], and breast cancer [35,36,37] as well as leukaemia [33,38,39,40] and non-Hodgkin’s lymphoma [33,41,42]. Less evidence has been reported on prostate [43], colorectal [44], liver [31], and paediatric cancer risk [45,46].

The aims of the study are to analyse the time trends in cancer incidence rates in the oil shale industry-affected areas in Estonia and compare those with national incidence rates, and time trends.

## 2. Materials and Methods

The descriptive registry-based investigation of cancer risk is part of the “Studies of the health impact of the oil shale sector—SOHOS” study series, which was instituted as the basis for the National Development Plan for the Use of Oil Shale, 2016–2030.

Population-based data on cancer cases were retrieved from the Estonian Cancer Registry for the years 1992–2015. Estonia has had a population-based cancer registry since 1968. Reporting of cancer cases to the Cancer Registry is mandatory for all physicians and pathologists in Estonia who diagnose and treat neoplasms. The data quality of the registry has been monitored for many years, and it meets all international quality standards [47].

When obtaining information about different neoplasms from the registry, we used WHO ICD-10 codes (internationally accepted practices for recoding ICD-9 into ICD-10 are in place [48]) [49]. We included cancer sites in the study that have previously been indicated as having relationships with different industrial activities like oil production: lung cancer (C34), kidney cancer (C64), bladder cancer (C67), breast cancer (C50), leukaemia (C91-C95), and non-Hodgkin’s lymphoma (C82-C85) [50,51,52,53]. Information on patient (gender, date of birth, nationality) and tumour characteristics (date of diagnosis) was extracted.

### 2.1. Exposure Assessment

**Figure 1:**
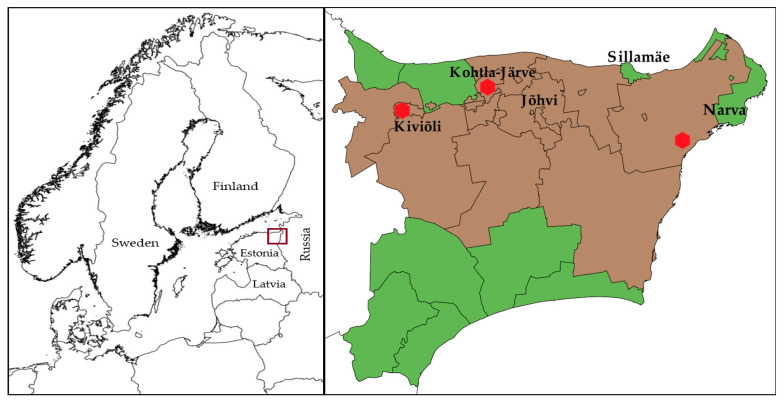
Map of Northern Europe showing the location of Estonia (left). Map of Ida-Viru County, Estonia, showing different municipalities (right). Municipalities under observation: oil-shale production and mining areas—brown, non-oil shale areas—green. The locations of shale oil extraction facilities are shown as red dots. The maps were made with QGIS 2.18 software. The administrative boundaries were provided by EuroGeographics and Estonian Land Board [56,57].

The main oil shale industrial area in Estonia is in Ida-Viru County in North-Eastern Estonia (Figure 1). The larger industrial cities in the area situate in the North of the Ida-Viru county. In the current study, the municipalities in Ida-Viru County were divided into those with a greater degree of exposure to industrial pollution (termed the “oil shale areas”) and less exposure to industrial pollution (termed the “non-oil shale areas”) based on Geological Survey of Estonia data on the mines, the locations of industrial sites, and Population Register data on people’s places of residence at the time of cancer diagnosis. Majority of the people living in the area are immigrants or their descendants, who moved there from all over the Soviet Union during the 1960s and the 1970s, making it a predominantly Russian-speaking region. Many of them speak still only Russian, making it a great barrier to live and work elsewhere in Estonia [54,55]. Therefore, exposure assessment based on the permanent residential location of the people was considered appropriate. “Oil shale areas” are the municipalities with industrial activity in shale oil chemistry and oil shale mining.

### 2.2. Statistical Analyses

Annual age-standardised cancer incidence rates for the period of 1992–2015 were calculated per 100,000 person-years using the population size in the administrative divisions as obtained from the Population Register of Estonia. Observed cancer sites in males and females are reported for all ages and in the age groups 0–19, 20–29, 30–39, 40–49, 50–59, 60–69, 70–79, ≥80 years. Subsequently, due to the small number of cases, we calculated three-year moving average incidence rates to reduce the fluctuating effect of different single years in the trend analysis.

Based on age-standardized morbidity rates (1992–2015), trend figures and linear trendlines were drawn for all observed cancer sites using Microsoft Excel (Office 365, Microsoft Corp, WA, USA). The paired t-test was used to compare the cancer incidence rate in the different areas (separately among males and females) using the statistical program Stata/SE 14.2 (StataCorp, College Station, TX, USA). A *p*-value of < 0.05 was considered statistically significant.

## 3. Results

The total registered numbers of observed cancer cases between 1992–2015 in Estonia and in Ida-Viru County were 51,525 and 6752, respectively. The mean ages at diagnosis of lung cancer, breast cancer, kidney cancer, bladder cancer, non-Hodgkin’s lymphoma, and leukaemia were 67, 62, 65, 70, 63, and 63 years in Estonia and 66, 60, 64, 68, 59, and 62 years in Ida-Viru County, respectively (Appendix A).

We also analysed the demographic composition of the study area and compared it to the rest of Estonia. During the study period, non-ethnic Estonians (73% Russians, 2% Ukrainians, 2% Belarusians) made up 81% of the population in Ida-Viru County, compared to 30% on average in Estonia (Table 1).

**Figure 2:**
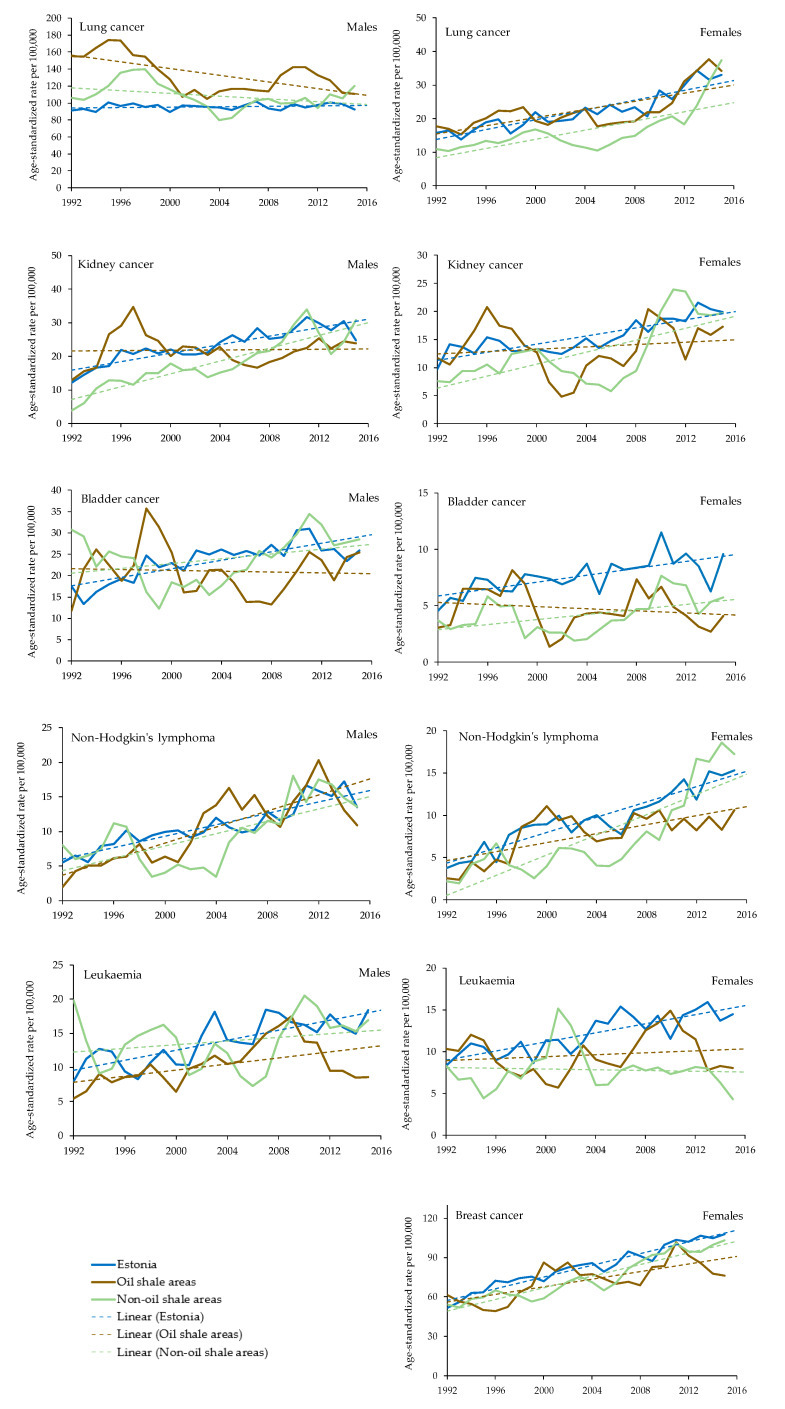
Age-standardized incidence rates for cancer sites in oil shale (brown line) areas, non-oil shale (green line) areas and Estonia overall (blue line). Cancer trendlines are represented with dashed lines.

Age-standardized incidence rates for cancer sites in oil shale areas, non-oil shale areas and Estonia overall are presented in Figure 2. The following is a description of these results for each cancer site.

### 3.1. Lung Cancer

The overall age-standardised incidence rate (ASIR) for lung cancer in males increased from 1992 to 2015 in Estonia by 1.5%; it increased in the non-oil shale areas by 13.3% (increase statistically significant, *p* < 0.05) and decreased significantly in the oil-shale area by 28.9% (*p* < 0.05). Even though the ASIR among males in the oil-shale area has decreased, the incidence rate for lung cancer remains higher than in non-oil shale areas and in Estonia overall (Figure 2).

For females, the ASIR for lung cancer in the oil shale areas increased from 17.6 per 100,000 person-years in 1992 to 34.2 per 100,000 person-years in 2015 (growth index +94%). The growth rates in Estonia overall and in the non-oil shale areas have been even larger: 109% and 181% (increase statistically significant), respectively.

### 3.2. Kidney Cancer

The ASIR in males for kidney cancer increased at an average of 3.7% per year in Estonia and 11.4% per year (trend also statistically significant) in the non-oil shale area. If at the beginning of the period (1992), the ASIR was higher in the oil shale areas, it stayed almost unchanged and was lower than on average in Estonia at the end of the study period in 2015. The incidence rate in females increased at an average of 4.0% per year over time in Estonia, 5.7% (*p* < 0.05) per year over time in the oil shale areas, and 6.1% per year in the non-oil shale areas (*p* < 0.05). The increase in non-oil shale areas was significantly higher compared to Estonia overall.

### 3.3. Urinary Bladder Cancer

The overall ASIR for urinary bladder cancer in males has increased during the observed period, with an average growth rate of 2.6% per year in Estonia and 6.7% in the oil shale areas, but it decreased by 7.2% in the non-oil shale areas. However, the cancer incidence rates are not statistically significant in different areas. The incidence rate in females has doubled in Estonia from 4.6 in 1992 to 9.6 per 100,000 person-years in 2015. The increase has been smaller in the oil shale areas, from 3.1 to 4.1, and in the non-oil shale areas, from 3.7 to 5.7, but the trend is still statistically significant.

### 3.4. Non-Hodgkin’s Lymphoma

Between 1992 and 2015, the ASIRs for non-Hodgkin’s lymphoma in males increased at an average of 3.7% per year in Estonia, 11.9% per year in oil shale areas, and 8.0% per year in the non-oil shale areas. Since 2006, the incidence rates in the oil shale areas have been slightly higher than in Estonia overall; however, there is no statistically significant difference between the areas. The incidence rates in females have also increased during the observed period, with an average growth rate of 8.5% per year in Estonia, 10.4% per year in the oil shale areas and 14.3% per year in the non-oil shale areas. However, there is a statistically significant difference between the areas, and the ASIR is lower in the oil-shale areas.

### 3.5. Leukaemia

The overall ASIR for leukaemia in males has increased during the last two decades (1992–2015) at an average of 5.4% per year in Estonia. The ASIR has been lower in the oil shale areas than in Estonia overall. The incidence rate in females has increased in Estonia from 8.3 in 1992 to 14.4 per 100,000 person-years in 2015 (growth index +73%). The incidence rates have significantly (*p* < 0.05) decreased from 1992 to 2015 both in the oil shale areas and the non-oil shale areas, from 10.3 and 8.3 cases per 100,000 person-years to 8.1 and 4.3 cases, respectively.

### 3.6. Breast Cancer

The ASIR in females for breast cancer increased at an average of 1.5% per year in the oil shale areas, 3.1% per year in the non-oil shale areas, and 3.5% per year over the period in Estonia overall. The incidence rates for females have been higher in Estonia overall and in the non-oil shale areas than in the oil shale areas.

## 4. Discussion

In this study, we investigated cancer incidence trends in oil shale industrial areas in Estonia. We focused on cancer sites that could be related to industry-specific pollutants in an industrialized region in North-Eastern Estonia. In the current study, we could see a higher ASIR only for lung cancer in the oil shale industrial areas as compared to the non-oil shale areas in the same county and in Estonia overall. Lung cancer is one of the leading causes of cancer-related morbidity and the most common cause of cancer-related mortality in males in Estonia [3]. Even though the incidence rates for lung cancer in males have decreased in oil shale areas, the incidence of lung cancer in those areas was higher during the study period compared to the Estonian average and the rest of Ida-Viru County. 

During an earlier analysis in 1989, Etlin et al. [18] presumed that cancer incidence would increase if ambient air pollution increased. However, since the 2000s, air quality has improved noticeably in the industrial region in Estonia (as reported by the monitoring station in Kohtla-Järve) [58]. During the same period, we could see the decrease in lung cancer incidence rates, and even though, we cannot justify the causal relationship with the current study design, the associations are possible. To improve the ambient environment, oil shale mining companies and shale oil extraction facilities have implemented new, more environmentally friendly production technologies [59]. However, as some recent studies have revealed, the environmental health effects on the respiratory system remain, as can be seen in increased prevalence of asthma among children [15].

Higher incidence rates of lung cancer could also be associated with occupational exposures. Several earlier studies [50,60,61,62,63,64] have shown that long-term occupational exposure to high levels of diesel exhaust is associated with an increased risk of lung cancer. As there are 16% of males in Ida-Viru County who are occupied in the oil shale industry, where diesel fuel is used to power the machines, they could therefore be at a higher risk of developing lung cancer. The indications of increased cancer morbidity risk could also be found from earlier biomonitoring studies among oil shale mining employees, who showed increased benzene metabolites excretion [65], increased porphyrin metabolism in lymphocytes (porphyrin associated with DNA increased 1.4 fold) [66], and an increased level of DNA damage as a higher number of DNA adducts [67]. As the Estonian Cancer Registry does not record occupational information, we could not analyse the effects of occupational exposures.

Still, we must admit that the trends in lung cancer incidence are as likely as to be influenced by past smoking prevalence than by environmental factors. Past smoking prevalence in males, particularly in populations with less education, has been high. According to the Estonian Health Behaviour Survey [68], the daily smoking prevalence in males in the 1990s was around 50% and has decreased since 2006. Therefore, some of the lung cancer incidence could be attributable to smoking habits, as a large number of males is exposed to direct or indirect contact with tobacco smoke daily in Ida-Viru County in the oil shale industrial areas [68]. The significant effect of smoking was also shown in earlier biomonitoring studies [67,69] in which the level of DNA damage in underground workers was significantly higher in smokers than in non-smokers. Silverman et al. [63] have also shown a combined effect as the cumulative exposure to tobacco smoke and diesel exhaust among males working in industries is increased threefold from that of non-smoking workers. We propose that the recent decreasing trend of lung cancer in males in the oil-shale areas could be the combined effect of positive changes in environmental exposure as well as past smoking habits.

In our study, we could also see increased lung cancer incidence rates in females in all areas. Aareleid et al. [70] have suggested that this increase in Estonia is attributable to an increased rate of female tobacco smoking. Estonian females have always had a lower prevalence of smoking than males, but smoking among females increased in the 1990s [68]. With a latency period of 20 years for lung cancer, the increase in tobacco smoking prevalence among females could explain the increasing trend of lung cancer incidence for females.

Another lung cancer risk factor prevalent in the area is radon. The main health damage caused by inhaling radon and its degradation products is lung cancer, which causes 3%–20% of all cases worldwide [71,72,73]. According to Petersell et al. [74] in 1/3 of the Estonian territory, including Ida-Viru County, the radon risk exceeds the limit considered safe for unrestricted construction, i.e., 50 kBq/m^3^. Due to the lack of personal-level data on radon exposure, and as exposure exceeding safe levels is prevalent in large areas, we could not take this effect into account. Nevertheless, the multiple and heterogeneous human exposures associated with these industrially contaminated sites make the assessment of possible lung cancer causes a challenging endeavour.

An aging population, better diagnostics, and increased screening have increased the overall incidence of cancer cases worldwide, and this is also the case in Estonia, including in Ida-Viru County [5]. The upward trend of breast cancer in Estonia is similar to other developed countries and could be due to increased screening and early detection as a result of the introduction of the Breast Cancer Screening program in 2002 for women 50–69 years [75]. However, the screening quality might vary, as Innos et al. [76] have shown a higher risk of advanced breast cancer diagnosis in the studied industrial area compared to two major Estonian cities, Tallinn and Tartu. Nevertheless, in contrast to earlier findings [33,45,77,78], no evidence of increased incidence rates of bladder cancer, kidney cancer, non-Hodgkin’s lymphoma, or leukaemia was detected among those living or working in an industrial area of Estonia. According to common environmental health practice and similar previous environmental impact studies [29,45], incidence is considered to be more appropriate for assessing relationships between morbidity and environment. Therefore, we used cancer incidence instead of survival, as cancer survival is more dependent on the functioning of the health care system and less dependent on the environment, and the findings might bias the actual situation.

Other explanations could be lifestyle-related factors such as obesity, hypertension, smoking, and alcohol consumption, which are quite widespread and unequally distributed. These factors definitely play a role in the emergence of cancer in the studied populations and could have masked the effect caused by oil-shale mining in the studied areas. Unfortunately, we did not have data on these possible confounders.

If we take a look at the public health status of the population in Ida-Viru County, it is in many ways worse than in the rest of the country (e.g., life expectancy is four years lower than in the capital area) [15,16,79,80]. Several studies [79,81] have indicated that lower education could be an obstruction to better health-related behavioural choices. According to the 2011 census [82], there were more people with lower education living in Ida-Viru County than elsewhere in Estonia. In 1998–2013, the highest age-standardised mortality rates for several causes of death were found in Ida-Viru County. Both males and females had higher mortality rates for HIV, injuries like accidental poisoning, exposure to narcotics (mainly illegal drugs), assault occurring mainly among young adults, and circulatory diseases that form from unhealthy lifestyle choices. As cancer is an aging-associated disease which likelihood increases with age [83], the cancer ASIR could therefore be lower in industrially contaminated areas.

As for the limitations of the current study, first, we admit that we did not have information for a number of factors that could possibly confound the association between exposure and outcome. We could not extract occupational data nor data on risk factors, as there is no collection of these data in the Cancer Registry. For the long time period under study, we did not have information about behavioural risk factors like smoking, alcohol use, excess body weight, and hypertension, data which may be available in the future by linking with other sources.

Second, there was no information on residents’ socio-economic situation, including their education or salaries. The earlier studies indicated slightly higher cancer incidence estimates for Russians than for Estonians, but this variation is likely to be attributable to exposure to specific etiological factors caused by differences in hygiene, smoking, and drinking [84] in Ida-Viru County.

Third, we could only address associations between cancer incidence and industrial pollution indirectly and lacked information about personal exposure such as dosage and time.

Fourth, the descriptive, registry-based study design may have been insufficient to capture findings associated with environmental and occupational risks. The design issues relevant to this type of investigation have been thoroughly examined by Elliott and Savitz [85], and a more detailed description of the limitations of ecological study design are available from Pirastu et al. [29,86], Savitz [87] and Wakefield [88]. The main constraint lies in the implicit assumption that residents in the industrial area experience similar exposures, while exposure variability is likely to be substantial due to many factors (e.g., distance of residence from polluting sources, occupation, lifestyle). Still, as suggested by Pirastu et al. [29], to enhance this study design, we selected a rather long time period, sufficiently large population size, and as the main strength of the study, we used validated and high quality data from the Estonian Cancer Registry, which has complete population coverage.

In the future, as many combined risk factors as possible should be included in a similar analysis. From the perspective of public health individual level data on confounding factors like smoking habits, lifestyle, socio-economic situation, migration, past exposures, occupations, and ambient conditions are essential to make regional and country wide health policy decisions.

## 5. Conclusions

In this study, the age-standardised cancer incidence rates for lung cancer in males were significantly higher in an industrial area in Estonia than in Estonia overall. One of the explanations for the higher lung cancer morbidity among males in Ida-Viru County could be associated with industrial pollution (oil shale production and shale oil extraction), yet adjustments on other lung cancer risk factors would be necessary to confirm this. However, in recent decades, the difference in lung cancer incidence rates has been decreasing, which could be associated with improved environmental quality in that region as one explanation. There were no significant differences between the rates of other studied cancer sites like urinary bladder, kidney and breast cancer, leukaemia, and non-Hodgkin’s lymphoma between those living in the industrialised areas and in Estonia overall. Prospective follow-up studies collecting exposure data at the individual level would be warranted but are not currently feasible due to the limited data available.

## Figures and Tables

**Table 1:** Demographic characteristics of the average population in Estonia and in Ida-Viru County in 1992‒2015.

Study Area	Average Population in Estonia	Average Population in Ida-Viru County	Average Population in oil Shale Areas	Average Population in non-oil Shale Areas
	n (%)	n (%)	n (%)	n (%)
**Total**	**1,376,819**	**175,765**	**67,230**	**107,452**
Gender				
Male	640,146 (46.5)	80,388 (45.7)	31,002 (46.1)	49,189 (45.8)
Female	736,673 (53.5)	95,377 (54.3)	36,228 (53.4)	58,263 (54.2)
**Age groups**				
0‒19	330,460 (24.1)	38,374 (21.8)	14,201 (19.4)	19,518 (19.3)
20‒29	192,720 (14.1)	21,654 (12.3)	9220 (12.6)	12,512 (12.4)
30‒39	193,075 (14.1)	24,160 (13.7)	8991 (12.3)	13,093 (13.0)
40‒49	189,606 (13.8)	26,418 (15.0)	11,071 (15.1)	15,482 (15.3)
50‒59	174,188 (12.7)	24,706 (14.1)	11,168 (15.3)	15,181 (15.0)
60‒69	148,577 (10.8)	21,373 (12.2)	8490 (11.6)	12,184 (12.1)
70‒79	101,568 (7.4)	13,987 (8.0)	7161 (9.8)	9732 (9.6)
80+	46,616 (3.4)	5093 (2.9)	2885 (3.8)	3366 (3.3)
**Ethnicity**				
Estonian	910,842 (69.6)	28,697 (19.3)	n/a	n/a
Russian	332,566 (25.4)	108,778 (73.3)	n/a	n/a
Ukrainian	23,005 (1.8)	3394 (2.3)	n/a	n/a
Belarusian	12,514 (1.0)	3325 (2.2)	n/a	n/a
Finnish	7690 (0.6)	1380 (0.9)	n/a	n/a
Other	22,264 (1.7)	2854 (1.9)	n/a	n/a

n/a—data not available for municipalities.

## Appendix A

**Table A1:** Age and ethnicity of cancer incidence of the studied cancer sites according to the gender in Estonia and in Ida-Viru County in 1992–2015.

Males																												
	Lung cancer				Kidney cancer				Urinary bladder cancer				Leukaemia				Breast cancer				Non-Hodgkin’s lymphoma				Total of studied cancer sites			
	Ida-Viru County		Estonia		Ida-Viru County		Estonia		Ida-Viru County		Estonia		Ida-Viru County		Estonia		Ida-Viru County		Estonia		Ida-Viru County		Estonia		Ida-Viru County		Estonia	
	n	%	n	%	n	%	n	n	%	n	%	n	%	n	n	%	n	%	n	%	n	%	n	%	n	%	n	%
**Total**	2283	65.1	14740	57.3	393	11.2	3549	13.8	414	11.8	3580	13.9	231	6.6	2114	8.2	10	0.3	105	0.4	176	5.0	1646	6.4	3507		25734	
**Age at diagnosis**																												
0‒19	0	0.0	2	0.0	5	1.3	29	0.8	1	0.2	3	0.1	19	8.2	136	6.4	0	0.0	0	0.0	7	4.0	60	3.6	32	0.9	230	0.9
20‒29	2	0.1	9	0.1	0	0.0	9	0.3	0	0.0	4	0.1	7	3.0	52	2.5	0	0.0	0	0.0	16	9.1	76	4.6	25	0.7	150	0.6
30‒39	5	0.2	69	0.5	9	2.3	72	2.0	8	1.9	34	0.9	5	2.2	59	2.8	0	0.0	1	1.0	14	8.0	99	6.0	41	1.2	334	1.3
40‒49	102	4.5	605	4.1	37	9.4	302	8.5	11	2.7	121	3.4	14	6.1	133	6.3	1	10.0	7	6.7	22	12.5	142	8.6	187	5.3	1310	5.1
50‒59	447	19.6	2876	19.5	96	24.4	799	22.5	69	16.7	478	13.4	42	18.2	313	14.8	3	30.0	14	13.3	25	14.2	259	15.7	682	19.4	4739	18.4
60‒69	925	40.5	5621	38.1	144	36.6	1153	32.5	132	31.9	1104	30.8	64	27.7	560	26.5	3	30.0	36	34.3	41	23.3	413	25.1	1309	37.3	8887	34.5
70‒79	659	28.9	4389	29.8	76	19.3	911	25.7	147	35.5	1245	34.8	63	27.3	617	29.2	3	30.0	32	30.5	41	23.3	427	25.9	989	28.2	7621	29.6
80+	143	6.3	1169	7.9	26	6.6	274	7.7	46	11.1	591	16.5	17	7.4	244	11.5	0	0.0	15	14.3	10	5.7	170	10.3	242	6.9	2463	9.6
**Ethnicity**																												
Estonian	544	23.8	9538	64.7	91	23.2	2422	68.2	105	25.4	2414	67.4	50	21.6	1496	70.8	4	40.0	68	64.8	41	23.3	1132	68.8	835	23.8	17070	66.3
Russian	1502	65.8	4169	28.3	254	64.6	897	25.3	268	64.7	951	26.6	148	64.1	471	22.3	5	50.0	27	25.7	121	68.8	383	23.3	2298	65.5	6898	26.8
Ukrainian	58	2.5	344	2.3	12	3.1	61	1.7	10	2.4	71	2.0	12	5.2	51	2.4	1	10.0	3	2.9	4	2.3	45	2.7	97	2.8	575	2.2
Belorussian	68	3.0	184	1.2	12	3.1	40	1.1	11	2.7	34	0.9	5	2.2	21	1.0	0	0.0	2	1.9	3	1.7	16	1.0	99	2.8	297	1.2
Finnish	24	1.1	106	0.7	8	2.0	23	0.6	1	0.2	13	0.4	1	0.4	12	0.6	0	0.0	0	0.0	2	1.1	8	0.5	36	1.0	162	0.6
Other	87	3.8	399	2.7	16	4.1	106	3.0	19	4.6	97	2.7	15	6.5	63	3.0	0	0.0	5	4.8	5	2.8	62	3.8	142	4.0	732	2.8
**Females**																												
**Total**	505	15.6	3910	14.8	337	10.4	2716	10.3	124	3.8	1345	5.1	226	7.0	2132	8.1	1868	57.6	14169	56.7	185	5.7	1669	6.3	3245		26391	
**Age at diagnosis**																												
0‒19	0	0.0	1	0.0	1	0.3	29	1.1	0	0.0	1	0.1	16	7.1	142	6.7	1	0.1	1	0.0	5	2.7	26	1.6	23	0.7	200	0.8
20‒29	2	0.4	6	0.2	0	0.0	6	0.2	0	0.0	3	0.2	2	0.9	38	1.8	9	0.5	71	0.5	3	1.6	44	2.6	16	0.5	168	0.6
30‒39	8	1.6	37	0.9	7	2.1	36	1.3	1	0.8	8	0.6	4	1.8	58	2.7	93	5.0	636	4.5	11	5.9	70	4.2	124	3.8	845	3.2
40‒49	14	2.8	141	3.6	20	5.9	111	4.1	8	6.5	32	2.4	9	4.0	116	5.4	308	16.5	2321	16.4	16	8.6	95	5.7	375	11.6	2816	10.7
50‒59	74	14.7	539	13.8	52	15.4	448	16.5	13	10.5	128	9.5	33	14.6	257	12.1	493	26.4	3570	25.2	31	16.8	195	11.7	696	21.4	5137	19.5
60‒69	153	30.3	1140	29.2	117	34.7	805	29.6	30	24.2	292	21.7	48	21.2	487	22.8	434	23.2	3534	24.9	45	24.3	406	24.3	827	25.5	6664	25.3
70‒79	162	32.1	1354	34.6	102	30.3	885	32.6	45	36.3	490	36.4	80	35.4	637	29.9	391	20.9	3065	21.6	51	27.6	535	32.1	831	25.6	6966	26.4
80+	92	18.2	692	17.7	38	11.3	396	14.6	27	21.8	391	29.1	34	15.0	397	18.6	139	7.4	1421	10.0	23	12.4	298	17.9	353	10.9	3595	13.6
**Ethnicity**																												
Estonian	112	22.2	2657	68.0	90	26.7	1862	68.6	25	20.2	961	71.4	45	19.9	1479	69.4	344	18.4	9472	66.9	46	24.9	1166	69.9	662	20.4	17597	66.7
Russian	335	66.3	1026	26.2	224	66.5	725	26.7	86	69.4	324	24.1	159	70.4	547	25.7	1361	72.9	4315	30.5	122	65.9	414	24.8	2287	70.5	7351	27.9
Ukrainian	13	2.6	55	1.4	4	1.2	37	1.4	4	3.2	14	1.0	3	1.3	21	1.0	46	2.5	237	1.7	6	3.2	31	1.9	76	2.3	395	1.5
Belorussian	17	3.4	38	1.0	4	1.2	17	0.6	3	2.4	8	0.6	6	2.7	18	0.8	52	2.8	189	1.3	7	3.8	22	1.3	89	2.7	292	1.1
Finnish	7	1.4	25	0.6	3	0.9	20	0.7	2	1.6	10	0.7	3	1.3	12	0.6	18	1.0	106	0.7	2	1.1	14	0.8	35	1.1	187	0.7
Other	21	4.2	109	2.8	12	3.6	55	2.0	4	3.2	28	2.1	10	4.4	55	2.6	47	2.5	300	2.1	2	1.1	22	1.3	96	3.0	569	2.2
